# Editorial: Additive or synergistic impacts of sleep, circadian rhythm disturbances and other modifiable risk factors on established and novel plasma biomarkers of Alzheimer's disease pathology

**DOI:** 10.3389/fnagi.2023.1168062

**Published:** 2023-03-14

**Authors:** Omonigho M. Bubu, Korey Kam, Ankit Parekh, Indu Ayappa

**Affiliations:** ^1^Department of Psychiatry, Healthy Brain Aging and Sleep Center, New York University Grossman School of Medicine, New York, NY, United States; ^2^Department of Population Health, New York University Grossman School of Medicine, Center for Healthful Behavior Change, New York, NY, United States; ^3^Division of Pulmonary, Critical Care and Sleep Medicine at the Icahn School of Medicine at Mount Sinai, New York, NY, United States

**Keywords:** sleep disturbance, circadian rhythm disorders, Alzheimer's disease, biomarker, plasma

Alzheimer's disease (AD) is a multifactorial and heterogeneous disease with multiple contributors to its pathophysiology, including sleep disturbances, disorders, and circadian rhythm abnormalities. Mounting evidence supports the relationship between specific sleep features, circadian rhythm abnormalities, and AD pathophysiology (Ju et al., 2017; Olsson et al., 2018; Lucey et al., 2019; Minakawa et al., 2019; Winer et al., 2019; Mander, 2020; Wang and Holtzman, 2020). Recent studies have shown that the pathologic features of AD, including Aβ, tau, and neurodegeneration, all result in specific deficits in local and global sleep and circadian expression that depend on the underlying brain regions affected (Ju et al., 2017; Olsson et al., 2018; Lucey et al., 2019; Minakawa et al., 2019; Winer et al., 2019; Mander, 2020; Wang and Holtzman, 2020). More importantly, sleep disturbances and circadian rhythm abnormalities tend to co-occur with other AD modifiable risk factors (e.g., aging, vascular risk factors, mood, physical activity, diet, and lifestyle), some of which are associated with established biomarkers for AD progression (Elias et al., 2003; Luchsinger et al., 2005; Kaffashian et al., 2011; Gottesman et al., 2017). What remains unexamined are the combined effects of sleep disturbances/disorders, circadian rhythm abnormalities, and other commonly co-occurring modifiable risk factors on the biomarkers of AD pathology (Langbaum et al., 2012; Glodzik et al., 2014; Bangen et al., 2015; Gottesman et al., 2017; Vemuri et al., 2017). Further, methods to examine the confluence of these exposures have been limited, with potential causal mechanisms linking these synergistic exposures to AD progression yet to be fully understood. This Research Topic aimed to more deeply examine and understand the interactions between sleep, circadian rhythm abnormalities, and modifiable risk factors on both established and, more importantly, novel plasma AD markers, including markers of inflammation and axonal integrity.

In our first paper, Carvalho et al. examined whether sleepiness, as measured by the Epworth Sleepiness Scale (ESS), is associated with other biomarkers of Alzheimer's disease (AD), axonal integrity, and inflammation, which may also contribute to neurodegeneration and cognitive decline. This was a cross-sectional analysis of data from 260 cognitively unimpaired adults (>60 years) from the Mayo Clinic Study of Aging. Participants had CSF quantification of AD biomarkers (Aβ42, p-tau, p-tau/Aβ42) in addition to at least one of the following biomarkers: neurofilament light chain (NfL), interleukin-6 (IL-6), IL-10, or tumor necrosis factor-α (TNF-α). Adjusting was performed for age, sex, APOε4 status, body mass index, hypertension, dyslipidemia, and prior diagnosis of obstructive sleep apnea. Higher ESS scores were associated with higher CSF IL-6 and NfL but not with the other CSF biomarkers. A sensitivity analysis, however, showed that ESS scores were associated with CSF p-tau/Aβ42 in amyloid-positive participants. The findings suggest synergistic associations possibly exist between sleepiness and amyloid levels on p-tau/Aβ42, and this, in turn, may contribute to vulnerability to sleep disturbance, which may further amyloid accumulation in a feed-forward loop process. Recently, others and ourselves have shown that vascular risk factors and disturbed sleep, respectively, each act synergistically with the Aβ burden to promote cognitive decline (Rabin et al., 2018; Bubu et al., 2020, 2021, 2022). In addition, the Aβ burden and neuroimaging evidence of CVD pathology show additive effects on cognition (Marchant et al., 2012, 2013; Park et al., 2014; Vemuri et al., 2015; Kim et al., 2016).

Xiong et al. have provided a timely review on recent advances in our understanding of how sleep and circadian disorders can influence Alzheimer's disease pathology. Firstly, the authors covered basic science investigations of how sleep and circadian disturbance can alter two well-known pathological hallmarks of AD, amyloid beta and tau, as well as lesser-known contributions from oxidative stress, blood–brain barrier leakage, and brain region susceptibility to sleep fragmentation. Secondly, they highlighted the potential of both non-drug interventions of sleep and circadian disorders, such as bright light and physical exercise, and the effect of more common sleep drug interventions such as melatonin, benzodiazepines, and DORAs. Importantly, the review noted that substantial progress has been made in our understanding of the basic mechanisms that control the biological clock and the neural circuits involved in sleep. However, we know very little about how these systems are affected in the brain in neurodegenerative diseases, especially as it relates to other AD modifiable risk factors including sex, APOE4 status, depression, and drug use.

We are delighted to publish a report by Nick et al. who have added to our field the finding of an important neuronal susceptibility to chronic sleep fragmentation. In this paper, the authors examined the role of hypocretin/orexin (HCRT) in hippocampal and locus coeruleus neuronal injury in response to the chronic fragmentation of sleep in mice with and without HCRT. They show that with sleep fragmentation, the presence of HCRT increases amyloid beta and decreases cholinergic axon projections to the hippocampus, while not influencing sleep disruption effects on locus coeruleus neurons. Their report identifies a molecular mechanism in sleep loss-induced neural injury in the hippocampus and provides a rationale to assess the role of HCRT antagonists to prevent such sleep loss-induced hippocampal injury.

Our final paper in this topic by Turner et al. determined the interactive associations of apolipoprotein e4 (APOE-e4) and obstructive sleep apnea (OSA) on biomarkers of Alzheimer's disease and examined for the racial/ethnic differences of these associations. This study utilized baseline data from 1,387 participants (mean age = 69.73 ± 8.32; 58.6% female; 13.7% Black/African American, 18.4% of the sample had sleep apnea, and 37.9% were APOE-e4 carriers) in the National Alzheimer's Coordinating Center Uniform Dataset (NACC UDS). The biomarkers of AD that were assessed included CSF Aβ_42_, hippocampal volume, and white matter hyperintensities (WMH). A performance on the Montreal Cognitive Assessment (MOCA) was used as a surrogate for cognition. The findings show independent associations of OSA and APOE-e4 with CSF Aβ_42_, WMH volume, and MOCA scores. OSA and APOE-e4 did not interact to affect amyloid pathology; however, in Black/African American subjects, OSA and APOE-e4 interacted with significant associations with WMH and hippocampal volumes. These findings bolster the need for further research exploring the combined effects of modifiable and fixed risk factors for AD, especially in Black/African American populations, where this interaction may partially mediate increased levels of risk.

Overall, this special topic section presents the evidence of the associations between sleepiness and neuronal injury markers, with sleepiness and amyloid levels showing possible synergistic effects on p-tau/Aβ42. It also presents evidence—using WT mice models induced with chronic fragmentation—demonstrating that under conditions of sleep fragmentation, hypocretin/orexin is essential for the accumulation of amyloid-β. Moreover, it presents evidence showing that OSA and APOE-e4 are interactively associated with WHM in Black/African Americans. More importantly, the review highlights the need for more studies that provide a more comprehensive understanding of the mechanisms by which specific neurodegenerative diseases and pathogenic proteins affect the circadian rhythm and sleep system, as well as the interactions linking sleep and the circadian rhythm system with potential causal mechanisms resulting in synergistic exposure effects on AD progression. This topic remains an open and active area of research because of the continued need to understand both how and when to modify identifiable risk factors, not only across the general population but also with appropriate care and precision among minoritized populations who may suffer from a combination of under-characterization and under-appreciation in terms of AD risk.

## Author contributions

All authors listed have made a substantial, direct, and intellectual contribution to the work and approved it for publication.
